# Post-wildfire soil bacterial MAGs and metagenome analysis

**DOI:** 10.1128/mra.00444-26

**Published:** 2026-07-10

**Authors:** Lola Allen, Allyson Sheneman, Maureen A. Morrow

**Affiliations:** 1 Department of Biology, State University of New York at New Paltz 14821, New Paltz, New York, USA; DOE Joint Genome Institute, Berkeley, California, USA

**Keywords:** soil microbiome, wildfire, metagenome-assembled genome

## Abstract

We compare the differences between bacteria in soil affected by a wildfire to an unaffected area from Minnewaska State Park, NY, located in the biodiverse northern Shawangunk Ridge. We detail our metagenomic sequencing data, relative abundance of bacterial phyla, and the taxonomic classification of three MAGs.

## ANNOUNCEMENT

The increased risk of wildfires has become a growing concern and has been affected by factors such as increased temperatures and limited water availability (1). Wildfires alter the soil bacterial communities (2), which in turn contribute to a variety of soil properties, including those that affect plant growth (3). To further our understanding of soil microbial populations during wildfire recovery, we performed metagenomic analysis of soil at the site of an August 2022 wildfire in Minnewaska State Park, NY, in the Shawangunk Mountains, a site of rich biodiversity (4).

Surface soil samples were obtained from the mixed pitch-pine oak forest in Minnewaska State Park on 4 November 2023. The ground contained a thin layer of duff over bedrock. The coordinates of the burned and unburned samples were (41.7228, −74.3618) and (41.7237, −74.3620), respectively. DNA was extracted using the DNeasy PowerSoil Pro kit (Qiagen) as per the manufacturer’s instructions (5 burned and 2 unburned, 0.25g each). Pooled DNA was concentrated using AMPure XP Beads (Beckman) according to the manufacturer's instructions with the following changes: beads to DNA volume ratio of 6:4 and elution with H_2_O extended to 10 minutes.

Samples were prepared for metagenomic sequencing at SeqCoast (Portsmouth, NH, USA) using the Illumina DNA Prep tagmentation kit (#20060059) with Illumina Unique Dual Indexes. Sequencing was performed on the Illumina NextSeq 2000 platform using a 300-cycle flow cell kit to produce 2 × 150 bp paired reads. PhiX control (1%–2%) was spiked into the run to support optimal base calling. Read demultiplexing, read trimming, and run analytics were performed using DRAGEN v4.2.7 (Illumina, default settings). The number of reads created was 63,503,400 (average length 147.701 nucleotides) for the burned sample and 52,133,656 reads (average length 148.4948 nucleotides) for the unburned sample.

**Fig 1:**
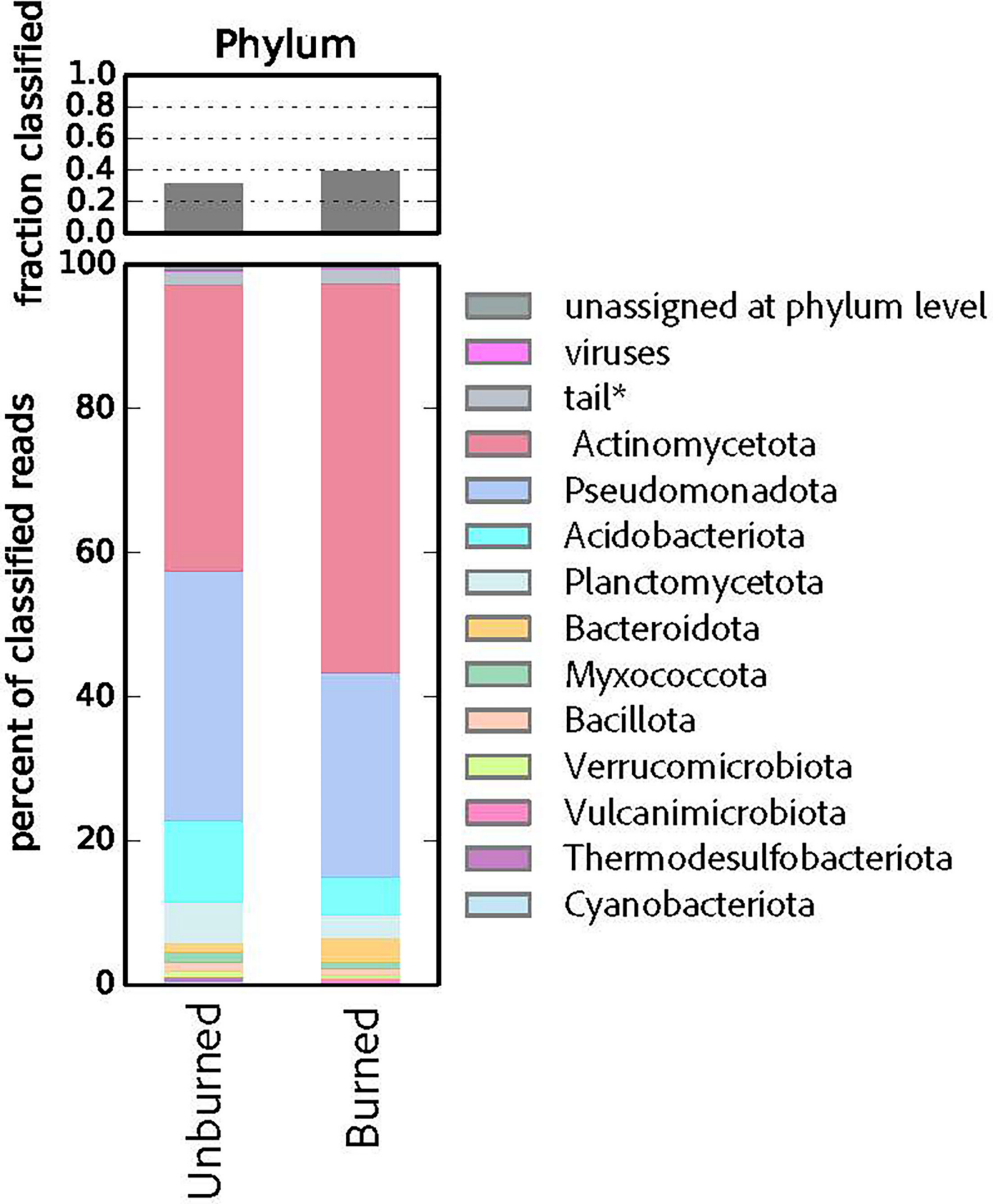
Kaiju (vl.9.0) phylum-level analysis of unburned and burned metagenome samples. *Tail belongs to a (non-viral) phylum with less than 0.5% of each taxon.

The reads were imported into KBase, and all analysis was performed using default settings unless otherwise indicated (5). Taxonomic classification of the reads was completed using Kaiju v1.9.0 (6), which showed the three most abundant phyla in both samples to be Actinomycetota, Pseudomonadota, and Acidobacteriota (Fig. 1). Consistent with several studies, the relative contribution of Actinomycetota increased and the relative concentration of Acidobacteriota decreased in the burned soil (7–9).

The reads were trimmed using Trimmomatic v0.36 (10) and assembled using metaSPAdes v3.15.3 (11), and the assemblies were assessed with QUAST v4.4 (12). The number of contigs was 21,421 (N_50_ 4,264) for the burned sample and 14,176 (N_50_ 3,299) for the unburned sample. The contigs were binned using MetaBAT2 v1.7 (13), and the genome quality was assessed with CheckM v1.0.18 (14). The results were filtered with the parameters of ≥93% complete and ≤3% contamination, which left three bins from the burned sample. The MAGs are described in Table 1 and were classified taxonomically using GTDB-Tk v2.3.2 (15). All MAGs are from the phylum Actinomycetota, consistent with the taxonomy results. One of the MAGs was assigned to the family Solirubrobacteraceae, while the other two MAGs were classified as the genus *Mycobacterium*. According to GTDB-Tk, all three potentially represent a new genus or species. These MAGs will contribute to our understanding of soil recovery after a wildfire, particularly for similar forests.

**TABLE 1:** Genome features and accession numbers of the post-wildfire metagenome-assembled genomes (MAGs)

Burned soil MAGs	Bin_6	Bin_7	Bin_9
Taxonomic classification*^a^*	d__Bacteria, p__Actinomycetota, c__Actinomycetia, o__Mycobacteriales, f__Mycobacteriaceae, g__Mycobacterium, s__	d__Bacteria, p__Actinomycetota, c__Actinomycetia, o__Mycobacteriales, f__Mycobacteriaceae, g__Mycobacterium, s__	d__Bacteria, p__Actinomycetota, c__Thermoleophilia, o__Solirubrobacterales, f__Solirubrobacteraceae, g__, s__
Completeness (%)^*b*^	96.19	96.09	93.02
Contamination (%)^*b*^	0.75	0.00	2.29
No. of contigs^*c*^	334	200	187
Size (Mbp)^*c*^	6	4.8	3.5
N_50_ (bp)^*c*^	29,420	50,855	30,238
GC content (%)^*c*^	65.70	65.50	66.30
No. of coding sequences^*c*^	5,765	4,694	3,392
Accession number	JBXFXA000000000	JBXFXB000000000	JBXTSK010000000

^*a*^ GTDB-Tk v2.3.2.

^*b*^ CheckM v1.0.18.

^*c*^ QUAST v4.4.

## Data Availability

The raw sequence reads are available under BioProject PRJNA1273997 and SRAs SRR33895690 and SRR33895689 (unburned and burned, respectively). The metagenome-assembled genome links are in Table 1. These Whole Genome Shotgun MAGs have been deposited at DDBJ/ENA/GenBank under accession numbers JBXFXA000000000, JBXFXB000000000, and JBXTSK000000000. The versions described in this paper are JBXFXA010000000, JBXFXB010000000, and JBXTSK010000000. The KBase analyses are available in a static narrative: https://kbase.us/n/250388/26/.
